# Clinical evaluation, motor performance and quality of life in patients affected by Soft Tissue Sarcomas undergoing surgical treatment: observational study

**DOI:** 10.3389/fonc.2025.1730371

**Published:** 2026-01-13

**Authors:** Andrea Demofonti, Marco Germanotta, Francesca Falchini, Arianna Pavan, Stefania Lattanzi, Laura Cortellini, Beniamino Brunetti, Stefania Tenna, Alice Valeri, Chiara Pagnoni, Roberto Passa, Michela Angelucci, Bruno Vincenzi, Rossana Alloni, Sergio Valeri, Irene Giovanna Aprile

**Affiliations:** 1 IRCCS Fondazione Don Carlo Gnocchi, Florence, Italy; 2 Operative Research Unit of Plastic-Reconstructive and Aesthetic Surgery, Fondazione Policlinico Universitario Campus Bio-Medico, Rome, Italy; 3 Operative Research Unit of Plastic-Reconstructive and Aesthetic Surgery, Universita` Campus Bio-Medico di Roma, Rome, Italy; 4 Operative Research Unit, Soft-Tissue Sarcomas Surgery Department, Fondazione Policlinico Universitario Campus Bio-Medico, Rome, Italy; 5 Operative Research Unit of Medical Oncology, Fondazione Policlinico Universitario Campus Bio-Medico, Rome, Italy

**Keywords:** human biomechanics, microsurgical reconstruction procedure, rehabilitation, robotics, soft tissue sarcoma

## Abstract

Soft Tissue Sarcomas (STSs) are rare malignant tumors characterized by a marked histological heterogeneity. Their standard of care is the surgical resection with adjuvant therapies, but these interventions induce sensorimotor impairments, pain, and a reduction quality of life. In this context, systematic evidence on its role in STS patients is currently lacking. This prospective, multicenter, observational study aims to evaluate the effects of a personalized rehabilitation protocol on clinical characteristics, motor performance, and quality of life in patients undergoing surgery for STS of the trunk and lower limbs. Patients will be recruited and assessed at four time-points: baseline (pre-surgery), post-surgery (within 7 days post-surgery), pre-rehabilitation (within 30 days post-surgery), and post-rehabilitation (within 90 days post-surgery). Rehabilitation will combine conventional physiotherapy with robotic technologies, delivered over two daily sessions for approximately 60 days. The assessments will include gait analysis, clinical scales, and patient-oriented questionnaires. The primary endpoint will be the improvement in functional status quantified in terms of Toronto Extremity Salvage Score differentiated for the Lower Limb, while secondary outcomes will include biomechanical parameters, pain, and quality of life. This trial will represent the first study quantifying the impact of rehabilitation in patients with STS, with the potential to generate novel prognostic factors and provide an evidence-based framework for future tailored rehabilitation protocols.

## 1 Introduction

Soft Tissue Sarcomas (STSs) are rare malignant tumors that account for 1% of adult solid cancers Gamboa et al. (1). Their annual incidence is estimated at approximately 24000 and 13500 new diagnoses in the European Union and the United States of America, respectively Gamboa et al. (1) Stiller et al. (2).

The clinical management of STSs is hindered not only by their rarity, but also by their biological diversity. Indeed, the World Health Organization recognizes more than one hundred histological and molecular variants, each displaying distinct pathological and clinical traits Jo and Fletcher (3). In addition, the STSs could develop in virtually any anatomical site, with the majority occurring in the limbs (60%), where the lower extremities are affected about three times more often than the upper ones Borghi and Gronchi (4), followed by the trunk (19%), retroperitoneum (15%) and head and neck (9%) Cormier and Pollock (5) Ebrahimpour et al. (6).

Although the STSs treatment generally considers both patient-related factors (e.g., age and comorbidities) and tumor-specific elements (e.g., histology, dimension and anatomical site), the combination of surgical excision and adjuvant therapies such as radiotherapy Haas et al. (7) or chemotherapy Maruzzo et al. (8) remains the reference standard Chowdhury et al. (9). The recommended radical resection requires excision of the tumor along with a margin of surrounding healthy tissue Casali et al. (10) creating large soft tissue defects which require additional reconstructive plastic surgery treatments Brunetti et al. (11).

Although effective from a reconstructive perspective, these operations compromise lower limbs sensorimotor functions inducing structural impairments (e.g., reduced joint movement, gait abnormalities), activity and social restrictions, and finally a reduction of Quality of Life (QoL) Gerrand and Furtado (12). Nonetheless, no studies have been conducted in this direction Andrews et al. (13).

Therefore, this study aims to deeply evaluate and objectively quantify the effects of a personalized rehabilitation protocol following STSs surgical treatment on clinical characteristics, sensorimotor performance, and QoL.

The paper is organized as follows: Section II describes the details of the experimental study, while Section III discusses the proposed clinical trial. The conclusions and future steps are exposed in Section IV.

## 2 Materials and methods

### 2.1 Study objectives

The main purpose of the study is to assess the effects of a personalized rehabilitation protocol on clinical status, motor performance, and quality of life in patients undergoing surgery for STS lower limbs.

To meet this objective, the following steps were planned:

- Identification of clinical characteristics and motor damage after STS surgical removal;
- Impact of perioperative treatments and surgery on patients QoL;
- Impact of personalized post-operative rehabilitation treatment on patients clinical characteristics, motor performance and QoL.

### 2.2 Study design and setting

The clinical investigation was designed as a prospective, observational, multicenter, uncontrolled study conducted on patients with STS localized in the trunk or in the lower limbs. It was approved by the Ethics Committee of the Fondazione Policlinico Universitario Campus Bio-Medico (Protocol number PAR 77.22 OSS), by the Ethics Committee Lazio 1 (Protocol number 420/CE Lazio 1) and registered on ClinicalTrials.gov (ID NCT06282237).

The study will be conducted at two centers: the Fondazione Policlinico Universitario Campus Bio-Medico as study sponsor, recruitment and surgery center, and the Centro Santa Maria della Provvidenza of the Fondazione Don Carlo Gnocchi in Rome as rehabilitation center.

**Figure 1:**
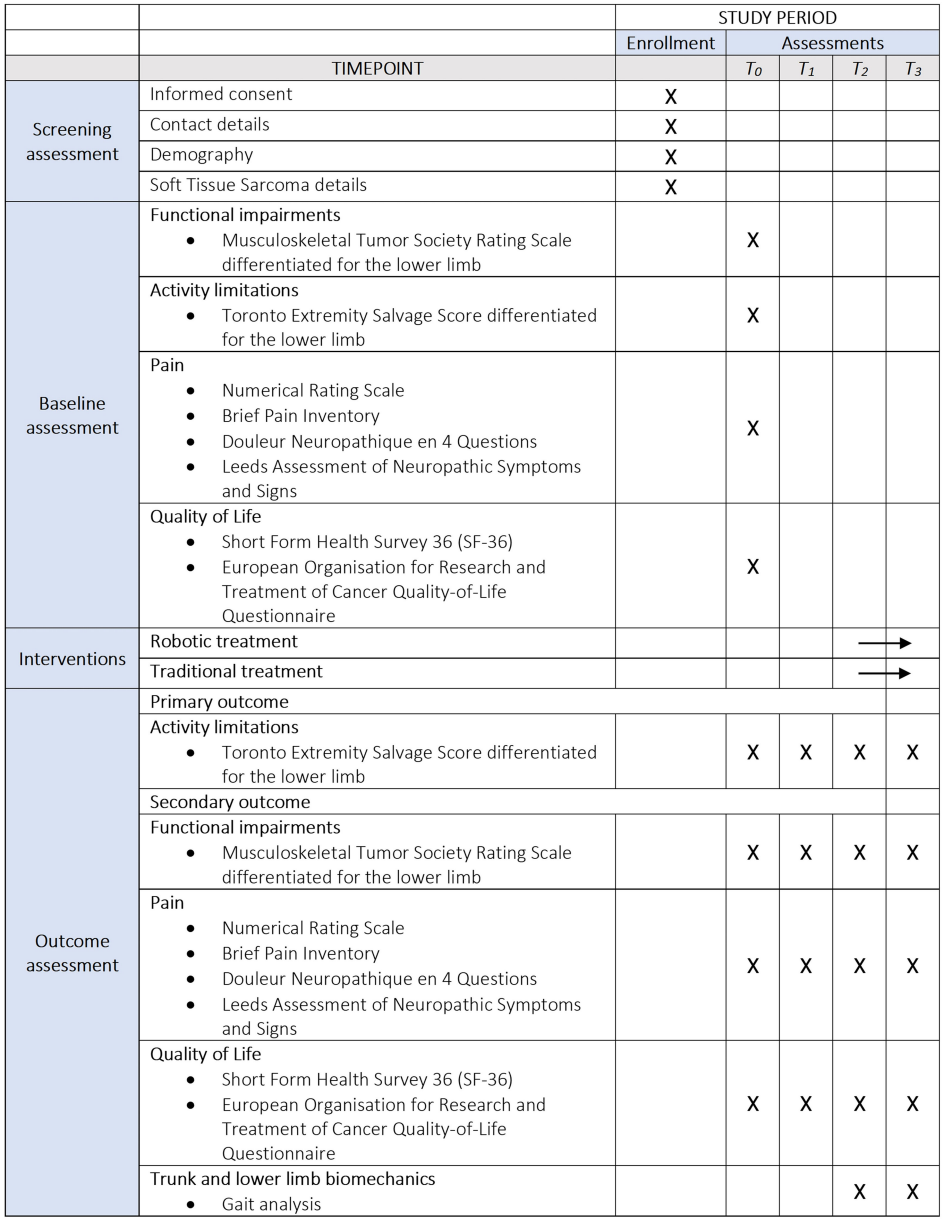
Standard Protocol Items: Recommendations for Interventional Trials (SPIRIT) schedule of enrollment, interventions and assessments. *T_0_* (within 7 days before surgery): baseline assessment; *T_1_* (within 7 days post-surgery): post-surgery assessment; *T_2_* (within 30 days post-surgery): pre-rehabilitation assessment; *T_3_* (within 90 days post-surgery): post-rehabilitation assessment.

The study is presented following the Standard Protocol Items: Recommendations for Interventional Trials (SPIRIT) Chan et al. (14) reported in **Figure 1**.

### 2.3 Study population

The patients’ screening will be carried out by a multidisciplinary team aiming to define the eligibility according to the following inclusion/exclusion criteria:

#### 2.3.1 Inclusion criteria

*i)* Age over 18 years; *ii)* i) patients affected by primary STS localized in the lower limb, in the trunk where a wide excision is needed or in the retroperitoneum whose resection or injury may impair lower-limb function such as the iliopsoas muscle, the lumbar plexus roots or the femoral nerve with curative intent; *iii)* defects with a diameter ranging from 10 cm to 15 cm.

#### 2.3.2 Exclusion criteria

*i)* Recurrent tumors; *ii)* metastatic diseases; *iii)* palliative surgery; *iv)* amputations.

### 2.4 Recruitment

The study will be proposed to the patients attending the specialist general surgery department for STS of the Fondazione Policlinico Universitario Campus Bio-Medico.

Before the recruitment, the patients will be screened to evaluate the fulfilment of the study’s inclusion/exclusion criteria. Once eligibility is confirmed, the principal investigator or designated representatives will obtain written informed consent. This procedure will be conducted only after patients have received clear and comprehensive information regarding the study objectives, methodology, assessments, potential benefits and any eventual risks. The consent form will specify the procedures for pseudonymizing collected data, ensuring patients’ confidentiality. During this process, the principal investigator or designated representatives will be available to address questions and provide clarifications, thereby ensuring that patients will have a full understanding of the study.

In addition, the recruitment center will keep a detailed screening log recording all patients evaluated for participation and documenting whether they were subsequently included or not.

### 2.5 Sample size

Based on the annual incidence of STS cases managed at the surgical center, a total of 50 patients will be recruited. Given the current absence of evidence regarding personalized rehabilitation in this population, the obtained results will be exploratory and will serve as a basis for determining the appropriate sample size in similar future trials.

### 2.6 Baseline assessment

Once the written informed consent is acquired, a baseline assessment will be conducted for each patient. Concerning patient’s demographic and clinical characteristics, the following data will be collected.

- Demographics (e.g., age, gender, height, mass);
- Past medical history;
- Tumor site;
- Histopathological tumor characteristics;
- Tumor staging;
- Information on adjuvant and/or neoadjuvant therapies and surgery;
- Medications taken;
- Functional impairments evaluated through the Musculoskeletal Tumor Society Rating Scale differentiated for the Lower Limb (MSTS-LL) Enneking et al. (15). This scale includes six domains regarding pain, functions, emotional acceptance, use of an external aid, walking, and lifting capabilities: it provides a total score ranging from 0% to 100% with higher values indicating better functional status;
- Activity limitations evaluated through the Toronto Extremity Salvage Score differentiated for the Lower Limb (TESS-LL) Davis et al. (16). It contains 30 questions assessing how difficult daily activities are (e.g., mobility, working, and wearing) execution on a scale from 0 (i.e., impossible to perform) to 5 (i.e., no difficulty). The final score ranges from 0% to 100% with higher values indicating better function;
- Pain evaluated through the Numerical Rating Scale (NRS) Downie et al. (17) and the Brief Pain Inventory (BPI) Cleeland and Ryan (18). The former quantifies pain in adult with a score ranging from 0 (no pain) to 10 (worst pain imaginable), the latter assesses pain intensity and its interference with daily activities with a score ranging from 0 to 10 with higher values indicating more severe pain and greater functional impairment. In addition, the neuropathic pain will be evaluated through the Douleur Neuropathique en 4 Questions (DN4) Bouhassira et al. (19) and the Leeds Assessment of Neuropathic Symptoms and Signs (LANSS) Bennett (20). The former includes 10 items: seven are related to patient-reported symptoms (i.e., burning, painful cold, electric shocks, tingling, needles, numbness, and itching) and three to clinical examination (i.e., hypoesthesia to touch, hypoesthesia to pinprick and pain provoked by brushing). Each positive answer scores 1 point, with a total score ranging from 0 to 10: a score of 4 or higher is indicative of neuropathic pain. The latter allows the discrimination between the nociceptive pain and the neuropathic one, combining subjective questions regarding symptoms (e.g., burning sensations, tingling, electric shocks) and objective sensory tests (e.g., changes in sensitivity to touch or pinprick). It provides a total score ranging from 0 and 24 with a value greater than 12 indicating a significant neuropathic component to the pain. From a general perspective, the use of the NRS, BPI, DN4 and LANSS will allow a granular characterization of the patient’s pain, including intensity, impact on activities and qualitative sensory descriptors;
- The perceived QoL evaluated through the Short Form Health Survey 36 (SF-36) Ware Jr (21) and the European Organisation for Research and Treatment of Cancer Quality-of-Life Questionnaire (EORTC QLQ-C30) Aaronson et al. (22). The former comprises 36 items evaluating eight health domains QoL (i.e., physical functioning, role functioning, bodily pain, general health, vitality, social functioning, role emotional and mental health) with a score ranging from 0 to 100 with higher values indicating a better QoL. The latter focuses on five functional domains (i.e., physical, role, emotional, cognitive, and social), QoL and eight symptom-related dimensions (i.e., fatigue, nausea and vomiting, pain, dyspnea, insomnia, loss of appetite, constipation, diarrhea, and financial difficulties) with each item scored on a scale from 0 to 100. Higher values in the functional domains and QoL indicate better well-being, whereas higher values in the symptom scales reflect a greater symptom burden.

### 2.7 Study intervention

Following recruitment for surgical treatment, a multidisciplinary team will define the optimal therapeutic approach taking into account patient-related factors (e.g., age, past medical history, and comorbidities) as well as tumor-specific characteristics (e.g., histology, size, and anatomical location). The team will also consider the eventual addition of radiotherapy and/or chemotherapy before or after the surgery as neoadjuvant or adjuvant therapies, respectively: the use of brachytherapy is not planned in the study. Subsequently, the patients will undergo STS radical resection to obtain negative margin classified as R0 in accordance with the guidelines of the Union for International Cancer Control Hermanek and Wittekind (23) and a reconstructive plastic surgery recovering the soft tissue defects.

Afterwards, at the rehabilitation center, all patients will undergo the same (i.e., no randomization) rehabilitation treatment consisting of postural steps, bed–wheelchair transfers, verticalization, ambulation without weight bearing on the operated limb or with partial bearing and ambulation with gradual weight bearing with or without aid Galluccio et al. (24).

The rehabilitative intervention will include two 50-minute sessions per day, six days per week for a total duration of approximately 60 days and it will imply both a robotic and conventional treatment.

As for the former, the recover of muscular strength, balance, proprioception and lower limb joints range of motion will be pursued using one or more of the following rehabilitative robots:

- G-EO System (Reha Technology, Olten, CH): end-effector robot characterized by a body weight support and two footplates placed on a double crank and a rocker gear system, inducing locomotor gait pattern;
- Lambda (Lambda Health System, Yverdon-les-Bains, CH): end-effector robot for rehabilitation of patients with lower limb neuromotor disabilities. The patient sits in a chair and is secured distally by two footplates supporting single and multi-joint movements in passive, assisted or active mode;
- Walker View (TecnoBody, Dalmine, IT): an auto-adaptive instrumented treadmill. The robot will support the patients’ body weight and will adapt the speed according to the patient’s residual motor capabilities. In addition, the patient will be immersed in a virtual reality environment that could enhance the engagement during the session;
- Hunova (Movendo Technology, Genova, IT): a stabilometric platform training the patients’ equilibrium and posture in a sitting or standing position.

In accordance with Italian standards on inpatient rehabilitation, the proposed approach will not be limited to robotic treatment but will also incorporate traditional one where the motor exercises will be performed or assisted by physical therapists, mostly in one-to-one sessions.

As reported in **Figure 1**, the following four evaluation sessions will be planned throughout the duration of the study:

- *T_0_* (within 7 days before surgery): an analysis of the patient’s demographic and clinical characteristics will be carried out in order to define the most suitable surgical approach. Then, a clinical survey composed of MSTS-LL, TESS-LL, NRS, BPI, DN4, LANSS, SF-36, and EORTC-QLQ-C30 will be adopted to evaluate the patient functional impairments, activity limitations during daily activities, pain and QoL at the beginning of the study;
- *T_1_* (within 7 days post-surgery): the aforementioned clinical survey will be submitted to the patient to evaluate the surgery’s short-term effects on functional impairments, activity limitations during daily activities, pain, and QoL;
- *T_2_* (within 30 days post-surgery): the clinical survey will be submitted to the patients to evaluate the clinical status before the beginning of the rehabilitation treatment. Moreover, the patients’ ambulation capabilities during free overground walking will be analyzed as soon as the patient is able to walk. Gait analysis will be conducted using the optoelectronic, marker-based BTS Smart D 500 system (BTS Bioengineering, Milan, Italy) within a calibrated acquisition volume of 5 × 3 × 2 m³. Before to each session, eight cameras will be mounted on tripods and geometrically calibrated; twenty-two 10-mm photo-reflective markers will be positioned on specific anatomical landmarks according to the Davis protocol Davis III et al. (25). Kinematic data will be acquired at a sampling frequency of 100 Hz. Kinetic parameters will be collected through two P-6000 force platforms (BTS Bioengineering, Milan, Italy) at a sampling rate of 1000 Hz. Surface electromyography will be recorded using eight FREEEMG sensors (BTS Bioengineering, Milan, Italy) applied to the following muscles of the operated limb: 1) Rectus Femoris responsible for hip flexion and knee extension, 2) Vastus Lateralis responsible for hip flexion and knee extension, 3) Vastus Medialis responsible for hip flexion and knee extension, 4) Biceps Femoris responsible for hip extension and knee flexion, 5) Semitendinosus responsible for hip extension and knee flexion, 6) Gluteus Maximus responsible for hip extension, 7) Tibialis Anterior responsible for ankle flexion, 8) Gastrocnemius Lateralis responsible for ankle extension. These muscles were selected because they are superficial and play a primary role in physiological gait Barbero et al. (26). Electrode placement will follow the recommendations of the Surface EMG for the Non-Invasive Assessment of Muscles guidelines Hermens et al. (27). Myoelectric signals will be sampled at 1000 Hz. Whether access to the anatomical landmarks or muscle bellies is limited due to wound healing, medical dressings or other clinical constraints, alternative strategies will be considered based on the patient’s condition and the requirement for accurate measurement. The markers may be positioned over bandages when necessary, and EMG sensors may be placed as close as possible to the target muscle belly while respecting clinical safety and feasibility. During each session, the patients will complete ten barefoot walking trials at a self-selected speed along an 8.0 m straight walkway. When walking aids (e.g., unilateral crutch, cane, or walker) will be required, the same aid will be consistently used across all assessment sessions to ensure standardization of testing conditions;

• *T_3_*(within 90 days post-surgery): the evaluations carried out in the previous session will take place in order to assess the impact of the personalized rehabilitation treatment and of the entire study on patients clinical characteristics, motor performance, and QoL.

The first two evaluation sessions will be carried out at the surgical center, while the remaining ones will be held at the rehabilitation center.

### 2.8 Intervention adherence

Since the patients will undergo surgical treatment, rehabilitation and eventually radiotherapy and/or chemotherapy during their inpatient stay across the two study centers, a high degree of adherence to the intervention is expected. To ensure both consistency and quality in the delivery of the rehabilitation program, adherence will be systematically monitored by an independent team of clinical professionals. This team will conduct periodic reviews of the treatment session logs to verify protocol compliance. In cases of deviations or risks of reduced adherence, corrective strategies will be implemented. Such strategies may include providing targeted refresher training sessions for the involved clinical staff, as well as delivering timely feedback to therapists to reinforce protocol fidelity and optimize patient engagement.

### 2.9 Complementary and supportive therapies

In accordance with Italian standards for inpatient rehabilitation, the proposed program will not be limited to robotic technologies (i.e., the end-effector robots G-EO System and Lambda, the instrumented treadmill with body weight support Walker View and the stabilometric platform Hunova) but will also incorporate conventional physiotherapy methods. Each patient will follow an individualized treatment plan focusing on the lower limbs and trunk with the aim of facilitating postural transitions, improving balance, and restoring gait. The intervention will consist of daily sessions of approximately 50 minutes, administered five days per week, and continued throughout the entire study period.

All the conventional and robotic rehabilitative sessions will be daily recorded by the therapists.

### 2.10 Outcome assessment

#### 2.10.1 Primary endpoint

The primary endpoint of the study will be the improvement of TESS-LL score between *T_0_*(baseline) and *T_3_*(end of the rehabilitation treatment).

#### 2.10.2 Secondary endpoint

The secondary endpoint of the study will be the improvement over time of the patient’s clinical status and trunk and lower limb biomechanics.

The former will be evaluated through the use of the presented clinical scales and patient-oriented questionnaires; the latter will be quantified through the aforementioned gait analysis. The parameters adopted to quantify the patients’ ambulation performance will be: *i)* the stance/swing phase, gait cycle duration, step length, stride length for both limb as well as the corresponding symmetry indices computed as described in Demofonti et al. (28). The step width, walking speed and cadence will also be evaluated regardless the limb side; *ii)* the trunk and both lower limb joints kinematics along the sagittal plane. Therefore the trunk, hip, knee and ankle flexion/extension will be evaluated and the related ranges of motion will be quantified; *iii)* the vGRF exerted on the ground by both limbs and the related peak values, loading rates and symmetry indices Demofonti et al. (28); *iv)* the myoelectric activity of the muscles of the operated limb and the related Root Mean Square values. The Co-Contraction Index values will be assessed for the agonist-antagonist muscle pairs RF/BF and TA/GL Infarinato et al. (29). The eventual presence of correlation between the gait indicators and the quality of life parameters will be evaluated.

During data analysis, the authors would consider a sub-group comparison on the basis of STS location (i.e., thigh anterior, medial or posterior compartment) or the resected muscles.

### 2.11 Study withdrawal

The patients may withdraw from the study at any time, without penalty, and without the need to provide a justification. In such circumstances, no additional study procedures will be carried out, while data collected prior to withdrawal will be retained and analyzed in accordance with the study protocol.

Discontinuation may also occur at the discretion of the principal investigator. In this case, the reason for withdrawal will be duly documented and the investigator will be responsible for promptly informing the patient and arranging an appropriate clinical follow-up outside the study, consistent with standard medical practice.

In the event of a drop-out, a replacement patient will be enrolled, if necessary, in order to ensure the achievement of the planned sample size.

### 2.12 Statistical analysis

A statistical analysis on outcomes measures will be conducted.

Descriptive statistics of the sample will include frequencies for categorical data, median and interquartile range for ordinal variables, and mean and standard deviation for continuous measures.

#### 2.12.1 Primary analysis

The primary endpoint will be assessed by comparing the TESS-LL score at *T_0_*and *T_3_*using a non-parametric test for paired samples (e.g., Wilcoxon signed-rank test). In addition, the primary endpoint will be used to classify the patients as responders (a change in TESS-LL score greater than 7 points) or non-responders. Subsequently, baseline clinical data that will be significantly different between responders and non-responders will be adopted as inputs for multivariate logistic regression models aiming to investigate potential predictors of recovery.

#### 2.12.2 Secondary analysis

The data obtained from the clinical questionnaires will be compared using parametric (e.g., ANOVA) or non-parametric (Friedman test) tests, as appropriate. The data obtained through gait analysis at *T_2_*will be compared with those at *T_3_*using parametric (e.g., t-test for paired samples) or non-parametric tests (e.g., Wilcoxon signed-rank test), according to the data characteristics and distribution.

The significance p level will be 0.05, and corrections will be executed in case of multiple comparisons among groups of data.

### 2.13 Safety evaluation

According to the study design and objectives, no adverse events directly due to the experimental procedures are expected. Potential risks are limited to the clinical treatments routinely required for patients with STS. The probability of damages remains minimal, as no invasive procedures or untested therapies are included in the protocol. All technologies employed are CE-marked and commonly used in clinical practice for patient’s rehabilitation Molteni et al. (30) Maciejasz et al. (31) Aprile et al. (32) Demofonti et al. (33) Lauretti et al. (34).

To further safeguard patients, every treatment session will be conducted under the direct supervision of qualified physiotherapists. Should an adverse event nevertheless occur, predefined safety protocols will be activated immediately. These include incident management procedures, provision of first aid, and, if required, the prompt involvement of emergency medical services. All adverse events will be documented and communicated by the principal investigator to the relevant regulatory authorities.

### 2.14 Ethics and dissemination

Upon obtaining informed consent, patient data will be pseudonymised and entered into a secure electronic database. Access to this registry will remain strictly controlled: only the principal investigator will retain the capacity to link identifying information with study codes, while collaborators with database access will not be able to re-identify participants. The correspondence between patient names and identification codes will be stored in a separate, password-protected file exclusively accessible to the principal investigator. This file will be permanently destroyed once data collection and quality control procedures are concluded. At that stage, the dataset will be rendered fully anonymized through the irreversible deletion of re-identification keys and, where necessary, the removal of potentially identifying variable combinations. In accordance with regulatory requirements, the anonymized dataset will be archived for ten years following study completion. After this retention period, the data will be re-evaluated to verify that anonymity remains preserved and may then be retained indefinitely in anonymized form.

The dissemination of study results will occur solely in anonymised form. Data will be reported through publications in peer-reviewed, high-impact journals and presented at national and international scientific conferences, meetings and symposiums, ensuring the protection of patients privacy.

## 3 Discussion

In the last decades, the adoption of robotic technologies in rehabilitation (e.g., end-effector and/or exoskeleton) has gained growing attention Molteni et al. (30) Maciejasz et al. (31). The adoption of such robots has been shown to be (at least) as effective as high-intensity physical therapy due to their ability to supply highly-intensive, repeatable, accurate, and patient-tailored movement therapy, while guaranteeing patient safety and unloading therapist workload with respect to traditional methods Lum et al. (35) Husemann et al. (36) Basteris et al. (37) Timmermans et al. (38) Mehrholz et al. (39).

Despite these promising results, the introduction of robots and high-technological solutions into current clinical practice is still hampered by several issues such as socio-economic barriers, ethics, and legal considerations Demofonti et al. (33). In addition, the available scientific evidence remains inconsistent and it does not adequately represent all the neurological pathologies inducing upper and/or lower limb sensorimotor disabilities. Since stroke is the third most common cause of disability Feigin et al. (40), the majority of studies have been conducted in post-stroke patients, whereas other conditions are scarcely represented. Moreover, the scientific literature provides results that could be regarded as contradictory Aprile et al. (41) Veerbeek et al. (42). This is due to multiple factors, and particularly to the considerable heterogeneity in terms of study duration, session frequency and treatment modalities Aprile et al. (41) Veerbeek et al. (42). Additionally, the limited number of patients treated in many studies represents a significant obstacle to the overall evaluation of robots’ effectiveness.

In this context, the present study will provide a novel and essential contribution addressing the role of rehabilitation in patients with STS. The rarity of this pathology [i.e., 1% of adult solid cancers Gamboa et al. (1)] has so far limited the availability of systematic clinical evidence, leaving a major gap in both scientific and clinical knowledge. This trial will represent a milestone, not only because of the ambitious recruitment target of 50 patients but also due to its mid-term intervention perspective (i.e., 3 months), which will ensure a more robust and reliable evaluation of rehabilitation outcomes.

The results of this work are expected to generate insights into the STSs effects on human biomechanics, pain and QoL. Moreover, considering factors such as tumor location, surgical approaches and additional clinical variables, the study will have a huge prognostic factor foreseeing long-term functional outcomes. Such evidence could serve as a baseline for the design and development of highly personalized rehabilitation protocols, ultimately setting new standards in the management of patients with STS.

Finally, a unique strength of the present study will lie in its strong multidisciplinary team, bringing together general and plastic surgeons, physicians, physiotherapists and engineers. This collaborative framework not only will ensure methodological rigor and clinical relevance but also it will pave the way for translational impact, bridging the gap between technological innovation and patient-centered care.

## 4 Conclusion

This study aims to deeply evaluate and objectively quantify the effects of a personalized rehabilitation treatment following STSs surgical treatment on patients clinical characteristics, sensorimotor performance and QoL.

Up to a maximum of fifty patients will be recruited and assessed at four time-points: baseline, post-surgery, pre- and post-rehabilitation. The rehabilitation will combine conventional physiotherapy with robotic technologies and it will be delivered over two daily sessions for 60 days. The assessments will include gait analysis, clinical scales and patient-oriented questionnaires.

The expected results will hold significant implications. First, they will provide objective evidence on the short- and mid-term functional outcomes of STS surgery. Second, the study will support the development of personalized rehabilitation pathways identifying prognostic factors related to tumor location, surgical approaches and patient characteristics. Third, the adopted structured methodology and multidisciplinary approach will serve as a reproducible model for future trials in similar rare and complex diseases.

Nonetheless, the study will be affected by limitations. The study period will be limited to three months, restricting the possibility of retrieving long-term recovery. The adopted approach will prevent the evaluation of emerging modalities such as telerehabilitation, which could improve accessibility and continuity of care. Furthermore, the lack of nutritional and genetic assessments will limit the capacity to explore biological determinants of recovery.

Future investigations should therefore extend follow-up, integrated digital and remote rehabilitation solutions and consider both genetic and nutritional assessment.
